# Type I Interferon-Mediated Skewing of the Serotonin Synthesis Is Associated with Severe Disease in Systemic Lupus Erythematosus

**DOI:** 10.1371/journal.pone.0125109

**Published:** 2015-04-21

**Authors:** Christian Lood, Helena Tydén, Birgitta Gullstrand, Cecilia Klint, Christina Wenglén, Christoffer T. Nielsen, Niels H. H. Heegaard, Andreas Jönsen, Robin Kahn, Anders A. Bengtsson

**Affiliations:** 1 Department of Clinical Sciences Lund, Section of Rheumatology, Lund University and Skåne University Hospital, Lund, Sweden; 2 Department of Laboratory Medicine Lund, Section of Microbiology, Immunology and Glycobiology, Lund University, Lund, Sweden; 3 AnaMar AB, Lund, Sweden; 4 Department of Clinical Biochemistry, Immunology and Genetics, Statens Serum Institut, Copenhagen, Denmark; 5 Department of Pediatrics, Clinical Sciences Lund, Lund University, Lund, Sweden; INSERM-Université Paris-Sud, FRANCE

## Abstract

Serotonin, a highly pro-inflammatory molecule released by activated platelets, is formed by tryptophan. Tryptophan is also needed in the production of kynurenine, a process mediated by the type I interferon (IFN)-regulated rate-limiting enzyme indoleamine 2,3-dioxygenase (IDO). The aim of this study was to investigate levels of serotonin in patients with the autoimmune disease systemic lupus erythematosus (SLE), association to clinical phenotype and possible involvement of IDO in regulation of serotonin synthesis. Serotonin levels were measured in serum and plasma from patients with SLE (n=148) and healthy volunteers (n=79) by liquid chromatography and ELISA, as well as intracellularly in platelets by flow cytometry. We found that SLE patients had decreased serotonin levels in serum (p=0.01) and platelets (p<0.0001) as compared to healthy individuals. SLE patients with ongoing type I IFN activity, as determined by an in-house reporter assay, had decreased serum levels of serotonin (p=0.0008) as well as increased IDO activity (p<0.0001), as determined by the kynurenine/tryptophan ratio measured by liquid chromatography. Furthermore, SLE sera induced IDO expression in WISH cells in a type I IFN-dependent manner (p=0.008). Also platelet activation contributed to reduce overall availability of serotonin levels in platelets and serum (p<0.05). Decreased serum serotonin levels were associated with severe SLE with presence of anti-dsDNA antibodies and nephritis. In all, reduced serum serotonin levels in SLE patients were related to severe disease phenotype, including nephritis, suggesting involvement of important immunopathological processes. Further, our data suggest that type I IFNs, present in SLE sera, are able to up-regulate IDO expression, which may lead to decreased serum serotonin levels.

## Introduction

Systemic lupus erythematosus (SLE) is an autoimmune rheumatic disease characterized by systemic inflammation and involvement of multiple organ systems including skin, joints and kidneys [1]. The inflammation is mediated by tissue-deposited immune complexes (ICs) causing complement activation, infiltration of immune cells and tissue destruction. ICs are phagocytosed by plasmacytoid dendritic cells (pDCs) and may, if containing nucleic acids, activate toll-like receptors (TLR)7 or TLR9 and promote production of type I interferons (IFNs) [2, 3]. Type I IFNs, in particular IFN-alpha, are increased in SLE patients and related to disease activity. A type I IFN signature with several type I IFN-regulated genes and proteins highly up-regulated is often seen in SLE. Type I IFNs are considered key cytokines in SLE pathogenesis due to potent immunomodulatory effects [4–6].

**Fig 1:**
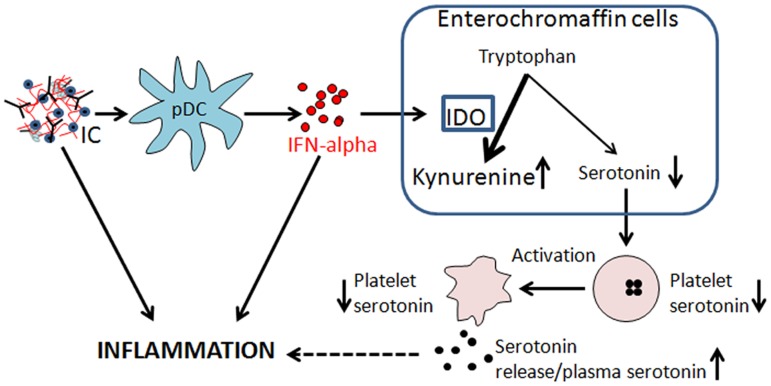
Summarizing figure of the main results and possible hypothesis. Nucleic acid-containing immune complexes (IC) are phagocytosed by plasmacytoid dendritic cells (pDC) and induce large amounts of interferon (IFN)-alpha. IFN-alpha up-regulates the expression of indoleamine 2,3-dioxygenase (IDO), increasing the conversion of tryptophan to kynurenine and limiting the availability of tryptophan for serotonin synthesis. The produced serotonin is taken up by the platelet close to the enterochromaffin cells. Upon platelet activation, serotonin is released and consumed by immune cells to amplify the inflammatory environment together with ICs and IFN-alpha.

Indoleamine 2,3-dioxygenase (IDO) is a type I IFN-regulated protein that is up-regulated in SLE patients [7–9]. IDO is the rate-limiting enzyme in the conversion of tryptophan into kynurenine. Kynurenine is an essential building block for several neuroactive metabolites, and skewing of the kynurenine pathways by inflammatory cytokines have been linked to central nervous system diseases [10–12]. By increasing the tryptophan conversion into kynurenine, IDO also limits synthesis of other tryptophan-dependent molecules, including serotonin [8, 13] (Fig 1). Serotonin is probably most known for its role as a signaling molecule in CNS synapses. However, most serotonin is produced in the periphery by intestinal enterochromaffin cells, where it is picked up by platelets and stored in dense granules [14, 15]. Upon platelet activation, serotonin is released locally to modulate hemostasis and inflammation [16–22]. Serotonin has been implicated as the driving force in establishing intestinal inflammation and serotonin receptor antagonists are able to block the inflammatory process in experimental animal models [23, 24]. Furthermore, serotonin has been shown to be involved in activation of monocytes and T cells, monocyte cytokine production, recruitment of neutrophils to inflammatory sites, extravasation of immune cells and regulation of type I IFN production through scavenging of ROS [18–22, 25], and those serotonin-mediated inflammatory effects have been implicated in rheumatic disorders [26–29]. Even though serotonin has been described to have profound inflammatory effects very little is known about its role in the pathogenesis of SLE and other chronic inflammatory diseases. To our knowledge very few attempts have been made to investigate serotonin in SLE and we found only four publications from the 80’s which all demonstrate decreased platelet serotonin levels in SLE [30–33]. The underlying mechanism behind the decreased levels of serotonin in SLE patients is however still unknown.

The aim of this study was to investigate peripheral levels of serotonin in SLE, association to clinical phenotype and the possible role of type I IFNs in regulation of serotonin synthesis. In brief we found that SLE patients had decreased platelet and serum levels of serotonin as compared to healthy individuals. The decreased serum levels of serotonin were highly associated with a severe SLE phenotype with involvement of nephritis and anti-dsDNA antibodies. Finally, a type I IFN-mediated skewing of the tryptophan metabolism and up-regulation of IDO, as well as ongoing platelet activation were identified as possible underlying mechanisms.

## Materials and Methods

### Patients

Patients with SLE (n = 148) were recruited at the Department of Rheumatology, Skåne University Hospital, Lund, Sweden. Healthy volunteers (n = 79) were age- and sex-matched to the SLE patients (SLE median 47 years, range 18–81 years, and healthy volunteers median 48 years, range 20–82 years, respectively and 85% and 87% women, respectively). An overview of the clinical characteristics is presented in Table 1. Disease activity was assessed using SLEDAI-2K [34]. All but two individuals fulfilled at least four American College of Rheumatology (ACR) 1982 criteria for SLE [35]. These two patients fulfilled three ACR criteria, had a clinical SLE diagnosis with at least two organ manifestations characteristic of SLE, autoimmune phenomena, and no other diagnosis that better could explain the symptoms. The following treatments were used in the SLE cohort at the time of blood sampling: corticosteroids (n = 98), hydroxychloroquine (n = 105), azathioprine (n = 32), mycophenolatmofetil (n = 20), methotrexate (n = 13), intravenous immunoglobulins (n = 2), non-steroidal anti-inflammatory drugs (n = 12), acetylsalicylic acid (n = 44), Warfarin (n = 23) and selective serotonin re-uptake inhibitors (SSRI) or other serotonin transporter antagonists (n = 21).

**Table 1:** Distribution of the American College of Rheumatology (ACR) 1982 classification criteria and patient characteristics for SLE patients (n = 148) included in the study.

Disease duration, median (range), years	11 (0–46)
SLEDAI score, median (range)	1.5 (0–18)
SLICC/ACR-DI score, median (range)	0 (0–8)
ACR criteria, median (range)	5 (3–10)
Malar rash %	52
Discoid rash %	20
Photosensitivity %	56
Oral ulcers %	24
Arthritis %	78
Serositis %	39
Renal disease %	33
Neurological disorder %	6
Hematological manifestations %	55
Leukopenia %	37
Lymphopenia %	24
Thrombocytopenia %	14
Immunology %	69
Anti-dsDNA antibodies %	59
ANA %	98

### Ethics statement

The study was approved by Lund University regional ethics board (LU-2010/544) and an informed written consent was obtained from all participants.

### Flow cytometry

Intracellular levels of serotonin in platelets were only measured in a subgroup of the recruited SLE patients (40/148) and healthy volunteers (64/79) due to logistic difficulties with fresh blood samples. For these platelet intracellular analyses, platelet rich plasma (PRP) was isolated from sodium-citrate blood through centrifugation for 10 min at 280 g. Isolated PRP was incubated with 2% paraformaldehyde for 45 min at room temperature. The incubation continued for 30 min at 37°C with the addition of 0.2% Triton X-100 to permeabilize the platelets. Between each of the following incubations, the platelets were washed once at 2500 g for 3 min in phosphate-buffered saline pH 7.4 (PBS). Anti-human serotonin antibodies produced in mouse (Dako, Glostrup, Denmark) were added and incubated for 15 min at 37°C followed by incubation with a FITC-conjugated rabbit anti mouse IgG antibody for 30 min at room temperature. The platelets were analyzed by flow cytometry on a BD Accuri C6 (BD, Franklin Lakes, NJ, USA). The results are presented as the median fluorescence intensity (MFI) ratio of the FITC fluorescence of the sample divided by the fluorescence of the secondary FITC-conjugated antibody alone.

### Interferon activity assay

Type I IFN activity was measured as previously described [36, 37]. Briefly, WISH cells (CCL-25; American Type Culture Collection, Manassas, VA, USA) were cultured for 6 h with patient serum after which lysis mixture (Panomics Inc., Fremont, CA, USA) was added. Cell lysates were analyzed on a Luminex 100 (Luminex Corporation, Austin, TX, USA) for mRNA expression of three house-keeping genes (GAPDH, PPIB, B2M) and six type I IFN-regulated genes (LY6E, MX1, OAS1, ISG15, IFIT1 and EIF2AK2) using the Quantigene Plex 2.0 assay as described by the manufacturer (Panomics Inc.). The type I IFN activity was calculated by the mean fold change of the type I IFN-regulated genes in WISH cells exposed to SLE serum as compared to unstimulated WISH cells.

### Measurement of serotonin, tryptophan and kynurenine

Serum was diluted 1/2 in PBS at a total volume of 200 μL with 90 μM 3-nitro-L-tyrosine (Sigma Aldrich) as an internal standard. For protein precipitation, trichloroacetic acid (0.4 M final concentration) was added and the samples centrifuged at 6000 rpm for 5 minutes. The upper 100 μL of the samples were saved for analysis of serotonin, tryptophan and kynurenine by liquid chromatography with ultra violet fluorescence detection (Agilent, Santa Clara, CA, USA) at Red Glead Discovery, Lund, Sweden. Tryptophan and serotonin levels were measured by fluorescence with excitation wavelength at 286 nm and emission at 366 nm. Kynurenine and 3-nitro-L-tyrosine were both detected by UV at 360 nm. The levels of serotonin were also measured with a commercially available ELISA according to the manufacturer’s instructions (LDN, Nordhorn, Germany) with similar levels as for the liquid chromatography (p<0.0001, r = 0.75, Spearman’s correlation). The 10^th^ percentile of the serotonin serum level in healthy individuals was used as a cut-off level for decreased serum levels of serotonin. Serotonin was also measured in EDTA plasma (isolated through centrifugation at 3000 g for 10 minutes at room temperature) with a commercially available ELISA according to the manufacturer’s instructions (LDN, Nordhorn, Germany). To estimate the relative amount of released serotonin, plasma levels (peripheral serotonin) were divided with serum levels (total serotonin). The 90^th^ percentile of the relative amount of released serotonin was used as a cut-off for increased peripheral serotonin.

### Analysis of IDO expression

WISH cells (CCL-25 ATCC, Manassas, VA, USA), were cultured in RPMI-1640 medium supplemented with 2.05 mM L-Glutamine, 10% fetal calf serum, 1% non-essential amino acid (vol/vol), 1 mM sodium pyruvate (PAA Laboratories GmbH, Pasching, Austria) and 100 μg/mL gentamicin (Invitrogen, Carlsbad, CA, USA) in a volume of 200 μL using Lab-Tek chamber slide (Nalge Nunc International, Naperville, IL, USA) for 3 h. In some experiments, anti-human interferon alpha/beta receptor (IFNAR) chain 2 (Clone NMHAR-2, pbl interferonsource, Piscataway Township, NJ, USA) blocking antibodies were added at a concentration of 5 μg/mL 3 hours prior to addition of 100 μL sera from three healthy individuals or eight SLE patients selected to have anti-ribonucleoprotein (RNP) antibodies. As a positive control, 650 U/mL of recombinant IFN-alpha (IntronA, Schering-Plough Company, Innishannon, Ireland) was used. After four days of incubation, cells were detached by 0.5 mM EDTA in PBS. WISH cells were fixed in 2% paraformaldehyde (Alfa Aesar, Karlsruhe, Germany) for 10 min and permeabilized in 0.25% Triton X-100 for 15 min, followed by addition of a phycoerythrin-conjugated anti-human IDO antibody (R&D Systems Minneapolis, MN, USA) for another 30 min at room temperature. IDO expression was analyzed by flow cytometry (Accuri C6, BD Pharmingen).

### Platelet activation

Platelet cell surface deposition of C4d was analyzed by flow cytometry as been described previously [38, 39]. For microparticle (MP) detection, flow cytometry was performed directly on EDTA plasma isolated through centrifugation at 3000 g for 10 minutes at room temperature [40, 41]. MPs were labeled with murine monoclonal anti-CD61-APC or the relevant isotype-matched control antibody. To reduce background noise, all buffers were filtered through 0.1 μm pore size filters (MiniSart HF, Sartorius Stedim Biotech S.A., Aubagne, France). Labeling was carried out by adding 5 μL heparin-sodium salt 10% w/v (Sigma-Aldrich 194 USP/mg dry basis) to 5 μL plasma followed by the addition of 5 μL pre-diluted anti-CD61-APC (IgG1k, final dilution 0.065 μg/mL, clone C7280, Dako, Glostrup, Denmark) or the isotype-matched control antibodies (IgG1k-APC, clone DAK-GO1, Dako). Then the suspension was diluted to 955 μL in low phosphate buffered saline with citrate (154 mM NaCl, 1.4 mM phosphate, 10.5 mM trisodium citrate, pH 7.4). The samples were analyzed using a FACSCalibur flow cytometer (BD, Franklin Lakes, NJ, USA) controlled by CellQuest software version 5.1.1 in the “high” flow rate mode. Flow rate was measured before each experiment. Both forward scatter (FSC) and side scatter (SSC), and the FL-4 fluorescence (anti-CD61) were recorded with logarithmic gain. μm Acquisition time was 60 seconds. MP gating was accomplished using 1 μm beads (Flow Cytometry Size Calibration Kit, Molecular Probes, Inc., Eugene, OR, USA) for setting upper limits in both FSC and SSC signals, and a lower limit was placed to exclude buffer noise as previously described [42]. Results from pilot studies clearly demonstrated that this gating strategy mainly captured MPs, whereas normal platelets were not included in the gate [40, 42]. Data analysis was performed using FlowJo software version 7.6.1 (Tree StarTreeStar, Inc., Ashland, OR, USA). Plasma concentrations (MPs/mL) were calculated on the basis of MP count per unit time, flow rate of the flow cytometer, and net dilution during sample preparation of the analyzed sample.

### Statistics

Spearman’s correlation test was used to analyze correlations between platelet intracellular levels and serum levels of serotonin. Variables not assuming Gaussian distribution were normalized through logarithms. Mann-Whitney U-test and ANCOVA was used for group analyses. Logistic regression analysis was used for all dichotomous variables. All analyses were adjusted for differences in age and sex when applicable. GraphPad Prism 5 (GraphPad Software, San Diego, CA, USA) was used for the figures and IBM SPSS Statistics 20 (Armonk, NY, USA) was used for the statistical calculations. A p-value <0.05 was considered statistically significant. All data utilized in the statistical analyses are provided in the Supporting Information S1 Table.

## Results

### Platelet and serum levels of serotonin are decreased in SLE patients

**Fig 2:**
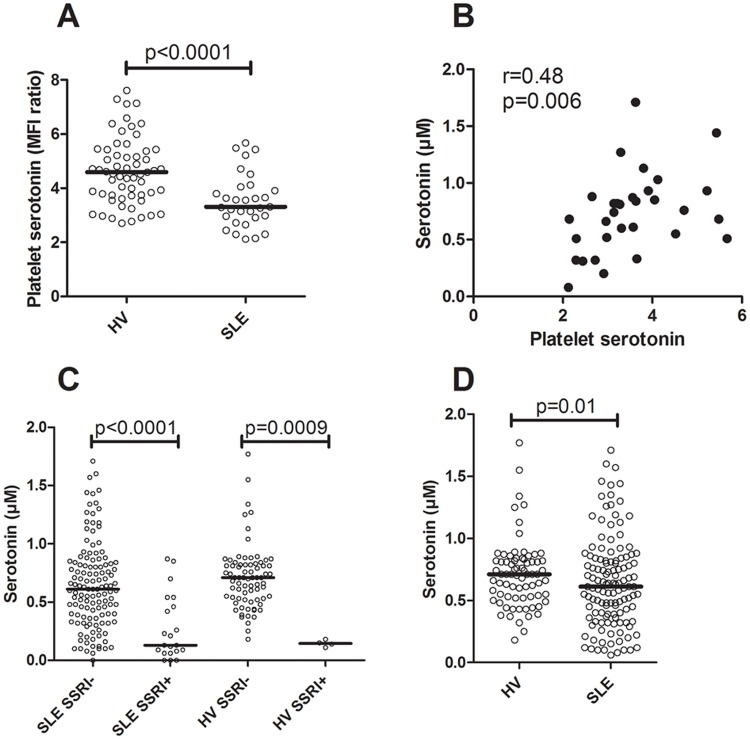
Serotonin levels in platelets and serum are dysregulated in SLE patients. A) Platelet intracellular serotonin concentration was analyzed by flow cytometry in a subset of the SLE patients and healthy volunteers (HV). B) Correlation analysis for serum and platelet levels of serotonin in SLE patients. C) Treatment with selective serotonin re-uptake inhibitors was associated with decreased serum levels of serotonin in both SLE-patients and HV. D) Serum concentrations of serotonin in HV and SLE patients. The bars represent the median value.

The platelet levels of serotonin were markedly decreased in SLE patients as compared to healthy volunteers (p<0.0001, Fig 2A), and correlated with the serum concentration of serotonin in both healthy individuals (r = 0.45, p<0.0001) and in SLE patients (r = 0.48, p = 0.006, Fig 2B). A fairly large proportion of the SLE patients (n = 21/148, 14%) used selective serotonin re-uptake inhibitors (SSRI) which highly affect the concentration of serotonin in serum (p<0.0001, Fig 2C). Among the healthy volunteers, only 4/79 (5%) used SSRI treatment and they also had a decreased serotonin concentration in serum (p = 0.0009, Fig 2C). In concordance with the intracellular levels of serotonin in platelets, SLE patients had decreased levels of serotonin in serum as compared to healthy individuals (p = 0.01, Fig 2D). Even though the use of SSRI was evenly distributed among the SLE patients and not increased in any of the investigated clinical phenotypes, patients (and controls) on SSRI treatment were excluded from all serotonin analyses, including the results depicted in Fig 2. However, to verify the robustness of the statistical models all analyses were also performed with the SSRI-treated patients included, but adjusted for, with similar results. None of the immunosuppressive treatments used by the SLE patients affected serotonin levels (data not shown).

### Low serum levels of serotonin are associated with a severe SLE phenotype

After establishing that serotonin levels were decreased in SLE, the association between serotonin concentrations and clinical phenotype according to ACR classification criteria was analyzed. SLE patients with nephritis had decreased serotonin levels in serum as compared to patients without this manifestation (OR = 3.1 (1.3–7.2), p = 0.01, Table 2). Furthermore, presence of anti-dsDNA antibodies, which is often seen in SLE patients with nephritis, were associated with decreased serum levels of serotonin (OR = 2.7 (1.2–6.5), p = 0.02, Table 2). No associations were found between serotonin levels and clinical disease activity at the time-point of blood sampling, possibly due to very few or no signs of clinical disease activity in most of the recruited patients. However, decreased serotonin levels were associated with complement consumption at the time-point of blood sampling (OR = 3.5 (1.4–8.4), p = 0.006, Table 2). In summary, decreased serotonin levels were seen in patients with a severe clinical phenotype with autoantibodies, complement consumption and nephritis.

**Table 2:** Low serum levels of serotonin are associated with severe SLE.

Manifestation	Positive (n = 127)	p-value^a^	Odds ratio (95% CI)^a^
Nephritis^b^	39	0.01	3.1 (1.3–7.2)
Anti-dsDNA^b^	76	0.02	2.7 (1.2–6.5)
Low C3/C4 levels^c^	35	0.006	3.5 (1.4–8.4)

^a^All analyses were adjusted for differences in age and sex.

^b^According to the 1982 ACR classification criteria.

^c^According to the SLEDAI-2K definition.

### The tryptophan metabolism is dysregulated in SLE patients

**Fig 3:**
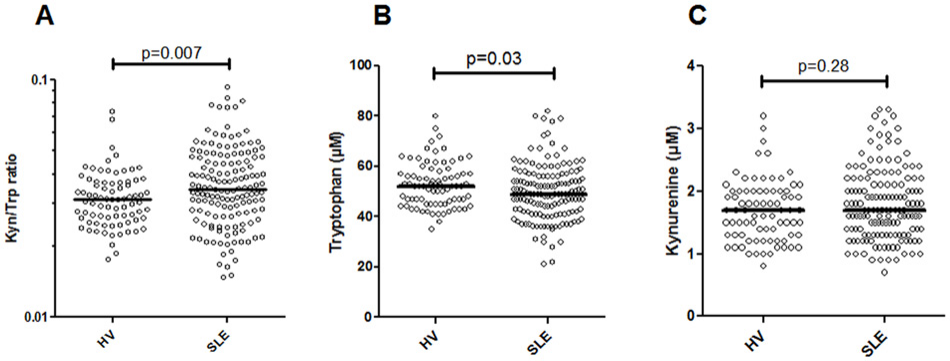
Dysregulated tryptophan metabolism in SLE patients. A) The activity of the enzyme indoleamine 2,3-dioxygenase (IDO) was indirectly measured as the ratio between kynurenine and tryptophan. The levels of B) tryptophan and C) kynurenine were measured by liquid chromatography.

Since total serum serotonin levels were decreased in SLE we next explored whether it was related to skewing of the tryptophan metabolism via IDO, as has been previously suggested [7–9]. Indeed, the activity of IDO, measured indirectly as the ratio between kynurenine and tryptophan, was increased in SLE patients as compared to healthy volunteers (p = 0.007, Fig 3A). SLE patients had a modest reduction in serum levels of tryptophan as compared to healthy individuals (p = 0.03, Fig 3B) whereas no differences were seen for the kynurenine levels (p = 0.28, Fig 3C). Thus, these findings suggest that SLE patients have a skewed tryptophan metabolism due to increased IDO activity.

### The IDO activity is regulated by type I IFNs in SLE patients

**Fig 4:**
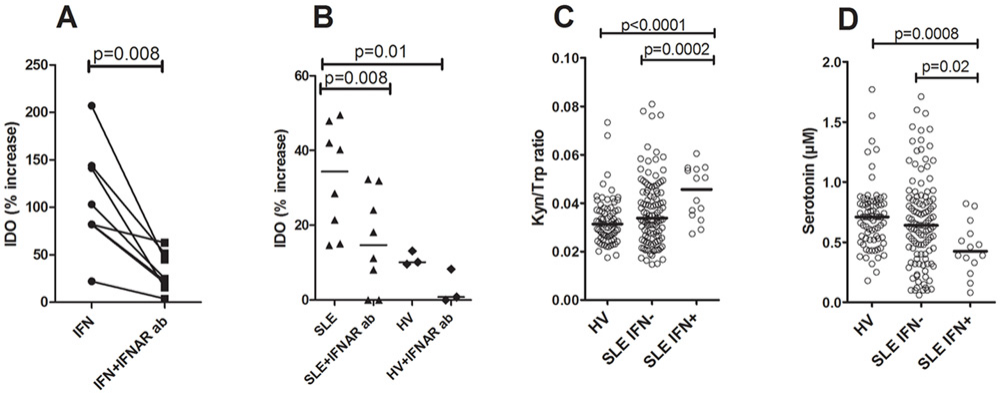
SLE sera increase IDO expression in a type I IFN-dependent manner. Endothelial cells (WISH cells) were incubated with A) recombinant IFN-alpha with or without pre-incubation with an IFN alpha/beta receptor blocking antibody (IFNAR ab) and IDO expression analyzed in permeabilized cells by flow cytometry. B) WISH cells were incubated with serum from SLE patients or healthy individuals with or without pre-incubation with an IFNAR ab. The results are presented as percentage increased IDO expression in median fluorescence intensity (MFI) as compared to non-stimulated cells. C) Patients with ongoing type I IFN activity (SLE IFN+) had increased IDO activity as compared to patients with no type I IFN activity (SLE IFN-) and healthy volunteers (HV). D) Patients with ongoing type I IFN activity had decreased serotonin levels as compared to patients with no type I IFN activity and healthy volunteers.

IDO activity has been described to be regulated by type I IFNs [13], a cytokine highly increased in SLE patients [43]. To investigate if type I IFNs were responsible for the increased IDO activity seen in SLE patients, sera were incubated with a type I IFN-sensitive cell line (WISH cells) and IDO expression was analyzed by flow cytometry. Physiological concentrations of recombinant IFN-alpha increased the IDO expression about 2-fold, and the IFN-alpha-mediated IDO expression could readily be inhibited by addition of an IFNAR-blocking antibody (p = 0.008, Fig 4A). SLE sera supported IDO expression to a higher extent than sera from healthy individuals (p = 0.01, Fig 4B), in a similar type I IFN-dependent manner as the positive control (p = 0.008, Fig 4B).

To assess if type I IFN-mediated regulation of IDO activity was seen in SLE patients, type I IFN activity was analyzed with an in-house cell reporter assay. SLE patients with high type I IFN production had increased IDO activity (kynurenine/tryptophan ratio) as compared to patients without type I IFN activity and healthy individuals (p = 0.0002, and p<0.0001, respectively, Fig 4C). Furthermore, SLE patients with high type I IFN production had a decreased serotonin concentration in serum as compared to SLE patients without ongoing type I IFN production as well as compared to healthy individuals (p = 0.02 and p = 0.0008, respectively, Fig 4D). Since type I IFNs clearly influenced serotonin levels through up-regulation of IDO, and have been associated with disease activity and severity in previous investigations [4], we re-analyzed the associations between disease manifestations and serotonin levels also taking into account differences in type I IFN activity. However, the association between decreased serotonin levels and disease severity, including development of nephritis, remained statistically significant even after adjusting for ongoing type I IFN activity, suggesting that decreased serotonin levels were associated with disease severity independently of type I IFN activity (data not shown). In all, our data suggest that type I IFNs, present in SLE sera, are able to up-regulate IDO expression, which may lead to decreased serotonin levels.

### Platelet activation contributes to reduce total serotonin levels

Although type I IFNs clearly were able to reduce serotonin synthesis through up-regulation of IDO, several mechanisms may operate in SLE to decrease the overall availability of serotonin in platelets, including platelet activation as depicted in Fig 1. Previous studies have demonstrated inverse correlations between low intracellular levels of serotonin in platelets and heightened levels of peripheral serotonin in plasma samples from SLE patients [33], suggesting that release of serotonin by activated platelets and subsequent consumption by surrounding immune or tissue cells may also reduce overall availability of serotonin.

**Fig 5:**
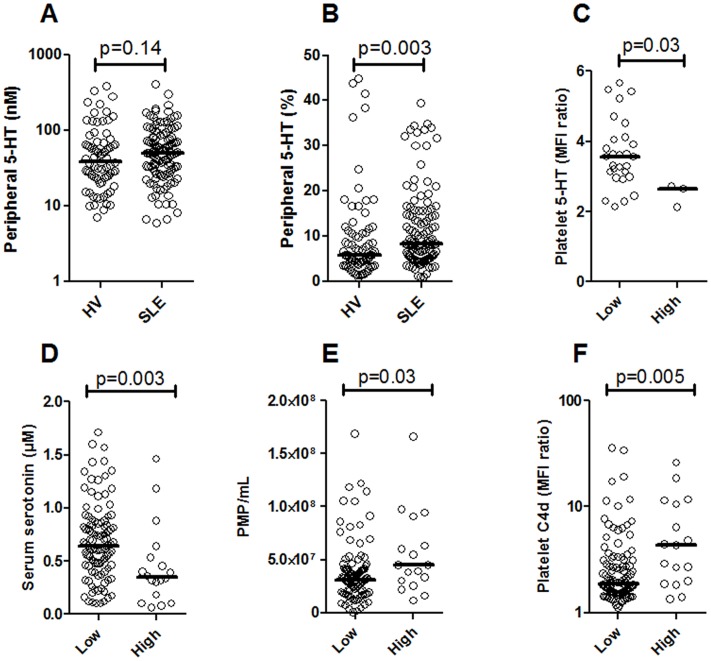
Decreased platelet serotonin levels are associated with platelet activation. A) Serotonin levels were analyzed in EDTA plasma samples and compared between healthy volunteers (HV) and SLE patients. B) To adjust for differences in total serotonin levels, the relative amount of peripheral versus total serotonin was calculated and compared between HV and SLE patients. SLE patients with a high relative amount of peripheral serotonin (high) was compared to SLE patients with low relative amount of peripheral serotonin levels (low) with regard to C) platelet intracellular serotonin levels, D) serum levels of serotonin, E) platelet microparticle (PMP) count in plasma and F) platelet activation assessed by platelet C4d deposition analyzed by flow cytometry.

In contrast to serum (total/platelet) levels of serotonin, plasma (peripheral) levels of serotonin were increased in SLE, although it did not reach statistical significance (p = 0.14, Fig 5A). However, taking into account the reduced overall availability of serotonin, the percentage of peripheral serotonin was highly increased in SLE patients (p = 0.003, Fig 5B). Compatible with previous study [33], high levels of serotonin in the periphery (plasma) were associated with decreased levels of serotonin both in platelets and serum (p = 0.03 and p = 0.003, respectively, Fig 5C and 5D). Furthermore, high levels of peripheral serotonin were associated with ongoing platelet activation as assessed by platelet microparticle formation (p = 0.03, Fig 5E) and platelet C4d deposition (p = 0.005, Fig 5F). In all, those data clearly demonstrate that also platelet activation participates in regulating platelet serotonin levels through releasing it into the periphery where it could induce inflammation in neighboring cells. However, the role of platelet activation in association to clinical manifestations is outside of the scope of the current investigation and will be studied in more details in future investigations.

## Discussion

Even though mainly thought of as an important neurotransmitter, the majority of serotonin is produced locally in the intestine supplying platelets with their serotonin stores enabling them to efficiently induce pro-inflammatory and pro-thrombotic signals upon activation and release of serotonin unto neighboring cells. In SLE, a disease characterized by systemic inflammation as well as markedly increased risk of developing thrombosis, decreased levels of serotonin in platelets have been described, but the underlying mechanism(s) was still lacking. In the current study, using a large cohort of SLE patients, we found decreased levels of serotonin in platelets from SLE patients as compared to healthy controls, also reflected by decreased levels of serotonin in serum. As illustrated in Fig 1, we hypothesized that the decreased serotonin levels found in SLE patients were dependent on deprivation of tryptophan, the building block of serotonin by rate-limiting enzyme IDO. Indeed, we were able to clearly demonstrate that SLE patients had increased IDO activity as measured by the shift in tryptophan and kynurenine, compatible with previous studies [8, 9]. Interestingly, even though IDO mainly catalyzes the conversion of tryptophan into kynurenine, several other IDO substrates have been described, including serotonin itself [44]. Thus, increased IDO activity may decrease serotonin content by increasing both tryptophan and serotonin turnover.

Next we investigated the cause of the increased IDO activity in SLE patients. IDO is well-known to be up-regulated in response to type I and type II IFNs, which are increased in SLE patients. Utilizing a reporter cell (WISH) we observed that SLE sera supported up-regulation of IDO expression to a higher extent than sera from healthy individuals. Furthermore, by using an IFNAR blocking antibody, we concluded that IDO was up-regulated in a type I IFN-dependent manner. This particular cell line is commonly used to analyze type I IFN activity in SLE sera due to its inability to respond to TLR7 and TLR9 ligands as well as low/absent expression of FcγRs [36, 45]. Not only did SLE sera support up-regulation of IDO in a type I IFN-dependent manner, but also SLE patients with ongoing type I IFN activity *in vivo* had increased IDO activity as well as decreased serotonin levels. Thus, we suggest that one explanation for the decreased levels of serotonin seen in SLE patients may be a type I IFN-mediated up-regulation of IDO (Fig 1). Up-regulation of IDO and depletion of serotonin is not specific for SLE, but has also been seen in patients with hepatitis C viral infections upon administration with recombinant IFN-alpha [13].

SLE is a very heterogeneous disease ranging from mild manifestations with rash to more severe inflammation in several organ systems including the kidneys and central nervous system. In our cohort, decreased serum levels of serotonin were strongly associated with a severe SLE phenotype, characterized by nephritis and anti-dsDNA antibodies, similar to previous studies in SLE [30, 31, 33, 46]. Importantly, the association was independent on type I IFN activity indicating a role of serotonin in the immunopathogenesis of SLE. The invert correlation with disease severity may at first seem contradicting considering the ample of literature supporting a role of serotonin in induction of inflammation, including activation and extravasation of immune cells, chemotaxis of neutrophils as well as regulation of type I IFN production [18–22, 25]. Furthermore, serotonin receptor antagonists are able to block the inflammatory process in several experimental animal models [19, 23, 24, 47]. Thus, serotonin has ample pro-inflammatory effects and therefore decreased serotonin levels would be expected to be associated with milder disease and not more severe disease as observed here.

However, even though serotonin has been ascribed some anti-inflammatory properties [48–50] possibly able to explain the inverse association with disease severity, the localization of serotonin is also important. Whereas the serum levels, e.g. platelet-stored serotonin, were decreased in SLE patients, the bioactive released serotonin found in plasma samples was increased. Importantly, we observed an inverse correlation between increased peripheral levels of serotonin and decreased platelet-stored serotonin, highly related to ongoing platelet activation. Thus, decreased levels of serotonin in serum, e.g. in the form of decreased platelet-stored serotonin, may reflect increased release and pro-inflammatory actions of serotonin, and likely cause a more severe phenotype.

In conclusion, SLE patients had a reduced serotonin level in serum and platelets related to severe disease phenotype, including nephritis, driven by immune complexes and type I IFN activation, but also representing other immunopathological processes which are central in SLE. As depicted in Fig 1, our data support the hypothesis of a type I IFN-mediated increase in IDO activity and subsequent deprivation of available tryptophan for serotonin synthesis in favor of kynurenine. However, other mechanisms, including platelet activation, may also participate to reduce peripheral serotonin levels, and this will be the subject of further studies.

## Supporting Information

S1 Table Supplemental database containing all relevant results obtained in the current study. (XLSX)
